# Dr. Leonard Koecker

**Published:** 1850-10

**Authors:** 


*Obituaries.*—We recently received a letter from Dr. Rodrigues, written at Paris, announcing the death of Dr. Leonard Koecker, of London. Dr. Koecker, as we have been informed, commenced practice in Baltimore, in 1807, but soon after moved to Philadelphia, where he acquired considerable celebrity as a practitioner. We believe he went to London about the year 1822, where he continued to exercise the duties of his profession until near the period of his death. As an operator, Dr. K. enjoyed a deservedly high reputation, and he has contributed very liberally to the literature of dental surgery.

